# Sitagliptin improves functional recovery via GLP‐1R‐induced anti‐apoptosis and facilitation of axonal regeneration after spinal cord injury

**DOI:** 10.1111/jcmm.15501

**Published:** 2020-06-22

**Authors:** Wen Han, Yao Li, Jiangting Cheng, Jing Zhang, Dingwen Chen, Mingqiao Fang, Guangheng Xiang, Yanqing Wu, Hongyu Zhang, Ke Xu, Hangxiang Wang, Ling Xie, Jian Xiao

**Affiliations:** ^1^ Molecular Pharmacology Research Center School of Pharmaceutical Science Wenzhou Medical University Wenzhou China; ^2^ Department of Orthopaedics The Second Affiliated Hospital and Yuying Children’s Hospital of Wenzhou Medical University Wenzhou China; ^3^ Key Laboratory of Combined Multi‐Organ Transplantation Ministry of Public Health School of Medicine The First Affiliated Hospital Zhejiang University Hangzhou China; ^4^ Engineering Laboratory of Zhejiang Province for Pharmaceutical Development of Growth Factors Biomedical Collaborative Innovation Center of Wenzhou The Institute of Life Sciences Wenzhou University Wenzhou China

**Keywords:** AMPK signalling pathway, axon regeneration, glucagon‐like peptide‐1 receptor, neurite outgrowth, sitagliptin, spinal cord injury

## Abstract

Axon growth and neuronal apoptosis are considered to be crucial therapeutic targets against spinal cord injury (SCI). Growing evidences have reported stimulation of glucagon‐like peptide‐1 (GLP‐1)/GLP‐1 receptor (GLP‐1R) signalling axis provides neuroprotection in experimental models of neurodegeneration disease. Endogenous GLP‐1 is rapidly degraded by dipeptidyl peptidase‐IV (DPP4), resulting in blocking of GLP‐1/GLP1R signalling process. Sitagliptin, a highly selective inhibitor of DPP4, has approved to have beneficial effects on diseases in which neurons damaged. However, the roles and the underlying mechanisms of sitagliptin in SCI repairing remain unclear. In this study, we used a rat model of SCI and PC12 cells/primary cortical neurons to explore the mechanism of sitagliptin underlying SCI recovery. We discovered the expression of GLP‐1R decreased in the SCI model. Administration of sitagliptin significantly increased GLP‐1R protein level, alleviated neuronal apoptosis, enhanced axon regeneration and improved functional recovery following SCI. Nevertheless, treatment with exendin9‐39, a GLP‐1R inhibitor, remarkably reversed the protective effect of sitagliptin. Additionally, we detected the AMPK/PGC‐1α signalling pathway was activated by sitagliptin stimulating GLP‐1R. Taken together, sitagliptin may be a potential agent for axon regrowth and locomotor functional repair via GLP‐1R‐induced AMPK/ PGC‐1α signalling pathway after SCI.

## 1 INTRODUCTION

Traumatic spinal cord injury (SCI) results in impaired neurologic function, such as severe motor and sensory dysfunction, that for many individuals is permanent and significantly impacts health, function, quality of life and life expectancy.^1^ Currently, there is no effective treatment for injured spinal cord, and the mechanisms that underlie recovery after SCI have rarely been definitively established. In a traumatic spinal cord injury, an external force causes immediate damage to neurons and axons at the injury site, resulting in a secondary neuropathological process that further aggravates neuronal damage.^2^, ^3^ Neuronal apoptosis is recognized as one of the most crucial secondary neuropathological processes underlying SCI, which eventually leads to significant expansion of the damage.^1^ Improving the neuronal intrinsic regenerative ability of central nervous system (CNS) by stabilizing microtubules can promote the axon regeneration and locomotor functional recovery after SCI.^4^, ^5^ Therefore, improving axon regrowth and neuronal survival remain the major prerequisite for regeneration and eventually functional recovery.

Glucagon‐like peptide‐1 (GLP‐1) is considered an incretin hormone when secreted by intestinal enteroendocrine L‐cell and a neuropeptide when released in the neural cells.^6^ GLP‐1 works by activating its specific receptor, GLP‐1 receptor (GLP‐1R), which belongs to the G protein‐coupled receptor family with seven‐transmembrane spanning.^6^ The GLP‐1/GLP‐1R signalling axis is best known for its effects on glucose homeostasis and facilitation of insulin signalling.^7^ GLP‐1 and GLP‐1R also are found in CNS. Beside the potential ability to regulate diabetes, the stimulation of GLP‐1R has been demonstrated to be neurotrophic and neuroprotective against neurodegenerative disease, such as Alzheimer's disease and traumatic injury as well as beneficial for neurogenesis in the CNS.^8^, ^9^, ^10^, ^11^ Evidences have supported that the activation of GLP‐1R has beneficial effects on neuronal cells survival, proliferation and differentiation, neurite outgrowth.^12^, ^13^, ^14^ Sun et al demonstrated abundant expression of GLP‐1R in primary spinal cord motor neurons^15^; GLP‐1R stimulation protected motor function through autophagy enhancement and suppressing neuron apoptosis in SCI,^16^ suggesting that activation of GLP‐1/GLP‐1R signalling axis plays a neuroprotective role in the SCI. Nevertheless, native GLP‐1 is rapidly degraded by the ubiquitously expressed enzyme dipeptidyl peptidase‐4 (DPP‐4) into the inactive forms which have very low affinity for the classical GLP‐1R.^6^, ^17^ The enzymatic activity of DPP‐4 is a major determinant of the biological activity of GLP‐1. Therefore, inhibiting the action of DPP‐4 is one way of increasing endogenous GLP‐1 levels and stimulating GLP‐1R to exert multiple effects in SCI.

Sitagliptin, a selective DPP‐4 inhibitor for increasing the half‐life of endogenous GLP‐1 and stimulating GLP‐1 receptor,^18^ is approved in more than 130 countries worldwide for the treatment of adult patients with type 2 diabetes (T2D).^19^ Sitagliptin is efficacious and safe drug for patients with type 2 diabetes mellitus because of its lower hypoglycaemic effect and low incidence of gastrointestinal complaints and reduced cardiovascular risk.^20^, ^21^ Besides its anti‐hyperglycaemia effect in T2D, sitagliptin can also exert roles of anti‐oxidative stress, anti‐inflammation and anti‐apoptosis in cardiovascular diseases,^4^ neurodegenerative disease and neural injury.^22^, ^23^, ^24^, ^25^, ^26^ Recently, increasing attention has been paid to the protective roles of GLP‐1R stimulation by GLP‐1 analogue or DPP‐4 inhibitors in SCI repairing. Actually, activation of GLP‐1R with GLP‐1 analogue exendin‐4 can significantly promote locomotion recovery in rats after SCI through the induction of autophagy and inhibition of apoptosis.^27^ Moreover, our previous studies have shown that another GLP‐1 analogue liraglutide improved locomotor functional recovery through stimulating autophagy and suppression of apoptosis via GLP‐1R after rat SCI.^21^

However, the potential neuroprotective benefits of sitagliptin have not been illustrated in SCI. In the present study, we aimed to assess the effects of sitagliptin on neurobehavioral outcome, axon regeneration and neuronal apoptosis in vivo and in vitro after SCI. We also examined the effects of sitagliptin on GLP‐1R. Finally, we explored whether the AMPK/PGC1α signalling pathway was involved in sitagliptin‐induced functional recovery following SCI.

## 2 MATERIALS AND METHODS

### 2.1 Animal usage and ethic statement

Adult female Sprague Dawley rats (6‐8 weeks old, 250‐300 g) were obtained from the Animal Center of the Chinese Academy of Science (Shanghai, China). The care and use of all animals is in line with the guidelines set by the National Institutes of Health of China. All the rats were reared under controlled conditions. Operations and animal experiments were approved by the Laboratory Animal Ethics Committee of Wenzhou Medical University.

### 2.2 SCI model and drug administration

SCI protocols in adult female Sprague Dawley rats under the aseptic conditions were as described previously. Briefly, rats were sedated with 8% (w/v) chloral hydrate (3.5 mL/kg, ip). Skin along the midline of back was cleaned and incised. Muscles were expanded, and a laminectomy was performed at T9‐T10 level. After laminectomy, the spine was stabilized and a moderate contusion on the thoracic spinal cord was performed using MACSIS/NYU Impactor (10 g forces, 3 cm height). Sham group being used as a control, received no damage after laminectomy. Afterwards, animals were orally treated with sitagliptin (10 mg/kg, saline) and then daily for up to 28 days after SCI. Another group was administrated with sitagliptin (10 mg/kg/d) and exendin9‐39 (1 μg/kg body weight, ip). Sham group was given equal saline. Subsequently, the rats were killed at 1, 3, 5, 7, 14 and 28 days after injury.

### 2.3 Primary cortical neurons culture

Primary cortical neurons were isolated from embryonic (E18) foetuses from pregnant Sprague Dawley rats and then seeded in poly‐D‐Lysine pre‐coated 12‐well plates in medium [80% (vol/vol) DMEM, 10% (vol/vol) fetal bovine serum (FBS) and 10% (vol/vol) F12]. 4 hours after plating, culture media were renewed with Neurobasal medium containing 2% B27 and 0.5 mmol/L L‐glutamine (GlutaMAXTM Supplement) and replaced every three days. DMEM, FBS and L‐glutamine were purchased from Gibco (California, USA) and Invitrogen (Carlsbad, CA, USA).

### 2.4 Tissue preparation

Animals were anesthetized by 8% (w/v) chloralic hydras (3.5 mL/kg, ip) at corresponding time points following SCI. Then, the hearts were perfused with normal saline. For haematoxylin‐eosin (HE) staining, Nissl staining and immunohistochemistry and immunofluorescence staining, 0.5 cm section of the spinal cord was dissected out, post‐fixed by 4% paraformaldehyde for 6 hours and then embedded in paraffin. Longitudinal or transverse sections (5 μm thick) were mounted on slides for subsequent staining. For Western blot, a spinal cord segment (0.5 cm length) at the contusion epicentre was dissected and stored at −80℃ immediately.

### 2.5 Western blot

Proteins from animals or primary cortical neurons were first quantified with BCA reagents, and 80 μg proteins were separated on 10% (w/v) gels and transferred onto PVDF membrane (Bio‐Rad, Hercules, CA, USA). Membranes were blocked with 5% (w/v) non‐fat milk (Bio‐Rad) in Tris‐buffered saline with 0.1% Tween‐20 (TBST) for 2 hours at room temperature and then incubated overnight at 4°C with primary antibodies [GLP‐1R (1:200, Santa Cruz, CA, USA); AMPK (1:500, Abcam, Cambridgeshire, England); ace‐tubulin (1:500, Abcam, Cambridgeshire, England); tyr‐tubulin (1:1000, Cell Signaling Technology, Danvers, MA, USA); Map2 (1:200, Cell Signaling Technology, Danvers, MA, USA) etc]. After being washed with TBST for three times, the membranes were treated with horseradish peroxidase‐conjugated secondary antibodies (1:10 000) for 1 hour. All result signals were visualized by ChemiDocXRS + Imaging System (Bio‐Rad, Hercules, CA, USA). All experiments were repeated three times to keep veracity.

### 2.6 Histology and immunofluorescence assay

Sections mounted on slides were prepared as described previously. Transverse sections for histological analysis were treated by HE staining and Nissl staining following the manufacturer's instructions. Bright field images were acquired using light microscopy. To assess acetylated, tyrosinated and growth cone in vitro, neurons were fixed and permeated in PHEM buffer (60 mmol/L Pipes, 25 mmol/L Hepes, 5 mmol/L EGTA, and 1 mmol/L MgCl_2_) containing 0.25% glutaraldehyde, 3.7% paraformaldehyde, 3.7% sucrose and 0.1% Triton X‐100 and quenched as above. For immunofluorescence, sections were treated with primary antibodies against the following proteins: MAP‐2 (1:500, Abcam, Cambridgeshire, England), GLP‐1R (1:100, Santa Cruz, CA, USA), GFAP (1:1000, Santa Cruz, CA, USA), Ace‐tubulin (1:1000, Cell Signaling Technologies, Danvers, MA, USA), Tyr‐tubulin (1:500 Sigma Aldrich, St. Louis, MO, USA), TOMM20 (1:400, Abcam, Cambridgeshire, England). The sections were washed with PBST four times for 3 minutes each and incubated with Alexa Fluor 568, Alexa Fluor 488 or Alexa Fluor 647 donkey anti‐rabbit/mouse secondary antibodies for 1 hour at 37°C. Next, the sections were washed with PBST for three times, then incubated with DAPI for 7 minutes, washed with PBST and sealed with a cover slip. For visualization of F‐actin, 4 U/mL rhodamine‐coupled phalloidin was used (Yeason, Shanghai, China). The images were captured by a confocal fluorescence microscope (Nikon, Japan). Three slides from different rats per group were used for histology and immunofluorescence assay, and five fields of visions were captured from a defined region of interest. And the positive area quantification has been normalized for tissue area analysed. The quantification of each density was performed by the Image J software.

### 2.7 ATP assay

The ATP‐GloBioluminometric Cell Viability Assay (Biotium, Hayward, CA, USA) was used to assess cellular ATP levels according to the manufacturer's protocol. Data were collected from five replicate wells for each experiment.

### 2.8 The terminal deoxynucleotidyltransferase (TdT) dUTP nick end labelling (TUNEL) assay

To measure apoptotic level, sections (4‐5 mm caudal and rostral) were obtained and TUNEL staining was performed at 7 days after SCI following the instructions. All images were captured using a Nikon ECLIPSE Ti microscope (Nikon, Japan). TUNEL‐positive cells were counted from 20‐30 random sections from three rats to get an average number in each group.

### 2.9 Locomotion recovery assessment

Basso‐Beattie‐Bresnahan (BBB) score and footprint analysis were performed to evaluate the restoration of hindlimb locomotor function of rats. In brief, BBB score was conducted at 0, 3, 7, 14 and 28 days. Rats were placed in an open experimental field and allowed to move freely for 5 minutes. The movement of rats hindlimbs was recorded by five trained investigators who were blind to the experimental conditions. Crawling ability was assessed by the BBB score ranging from 0 (complete paralysis) to 21 (normal locomotion). At the time point of 14 days after injury (dpi), the footprint analysis was performed by dipping the animal's posterior limb with red dye and fore limb with blue dye. All of the rats were tested in a confined walkway (about a 10 cm wide by 100 cm long white paper), and then the hindlimb motion trajectories printed on the white runway were collected.

### 2.10 Statistical analysis

All data were expressed as the means ± standard error of the mean (SEM). Differences between groups in BBB scores were analysed with one‐way analysis of variance (ANOVA) followed by Tukey's multiple comparison test. Statistical analyses of other data were performed using one‐way ANOVA. All statistical analyses were conducted using statistical software GraphPad Prism version 5 for Windows. Differences were suggested to be statistically significant when *P* < 0.05.

## 3 RESULTS

### 3.1 Sitagliptin promotes functional recovery and reverses neurological deficit after SCI

To assess the therapeutic role of sitagliptin in SCI repairing, rats were randomly assigned to three groups, including sham group, SCI group and SCI+ sitagliptin group. BBB score as well as footprint analysis were applied to evaluate locomotor recovery after SCI. The results showed that BBB scores of both of SCI surgery group were distinctly decreased compared with sham group, but with no significant difference between the two SCI group at early stage (1, 3, 5 days after injury, dpi). However, compared with SCI group, sitagliptin treatment group exhibited a significant functional improvement at 7, 14, 28 dpi (Figure S1B‐E). Furthermore, the footprint test at 14 dpi also verified the therapeutic effects of sitagliptin after SCI. Sitagliptin treatment group performed a coordinated crawling and tail lift and slight staggering gait whereas SCI group still dragged their hind limbs (Figure S1F). Results above indicate that sitagliptin contributes to locomotor functional recovery after SCI. We then examined the effect of sitagliptin on nerve growth using Nissl staining and HE staining. The ventral motor neurons (VMN) after SCI lost explicitly and sitagliptin administration notably reversed this trend (Figure S1G‐H). This result is consistent with that of HE staining. Compared with the sham group, SCI group exhibited a significant damage of central grey matter and peripheral white matter and a largest proportion of cavity (green dash line), while sitagliptin‐treated group remarkably reduced tissue loss and cavity formation (Figure S1I‐J). Taken together, sitagliptin has neuroprotective effects on both neuron survival and functional recovery after SCI.

### 3.2 Sitagliptin protects against neuronal apoptosis after SCI

**FIGURE 1:**
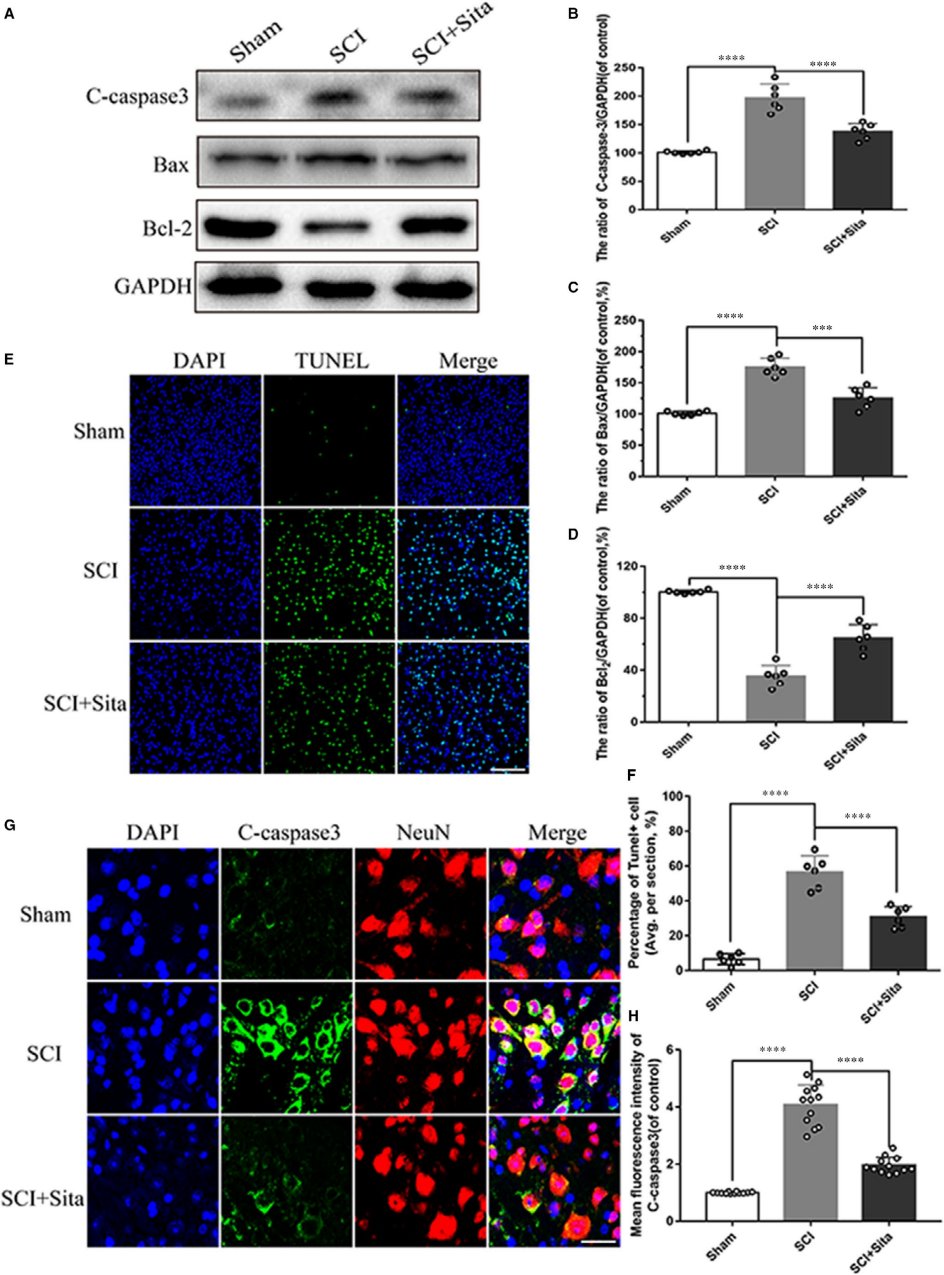
Sitagliptin protects neurons against apoptosis after SCI. A‐D, Western blot and the quantitative analysis of cleaved caspase‐3, Bax and Bcl‐2 in rats of each group at 7 d after SCI. GAPDH was used as a loading control and for band density normalization. E‐F, TUNEL staining (green) and the quantitative assessment of TUNEL‐positive cells from six independent sections from injured spinal cord in each group at 7 dpi, scale bar = 100 μm. G‐H, Co‐immunofluorescence for cleaved caspase‐3 (green) and NeuN (red) and the quantitative assessment of the fluorescence intensity of cleaved caspase‐3 in neurons in each group, scale bar = 20 μm. All data are presented as the mean values ± SEM, n = 6 per group. (The ‘n’ in the whole text means the number (or replication) of samples (rats or cell treatment) in each group). Sham is used as a control. ***P < 0.001, ****P < 0.0001 vs SCI group

To confirm the significance of sitagliptin after SCI and to explore the mechanism of neuroprotection of sitagliptin following SCI, we designed a timeline as shown in Figure S1A. Cell apoptosis occurs at the beginning of the injury and sustains in the entire recovery process. To determine whether sitagliptin has ability to decrease apoptosis caused by acute SCI, Western blot analyses for Bax, Bcl‐2 and cleaved caspase‐3 were carried out at 7 dpi. Compared with the sham group, acute SCI increased the pro‐apoptotic proteins Bax and cleaved caspase‐3 levels and reduced the anti‐apoptotic protein Bcl‐2, which were significantly reversed by sitagliptin (Figure 1A‐D). These results were verified by TUNEL staining and immunofluorescence staining. The number of TUNEL‐positive cells apparently rose after SCI, while administration of sitagliptin resulted in a clear fall in apoptotic activity (Figure 1E‐F). Similarly, immunofluorescence staining illustrated a prominent increase of the cleaved caspase‐3‐positive neurons in the SCI group, but that reduced with sitagliptin administration (Figure 1G‐H). Collectively, these data indicate that sitagliptin can inhibit neurons apoptosis after SCI.

### 3.3 Sitagliptin stabilizes microtubule and facilitates axon restoration after SCI

**FIGURE 2:**
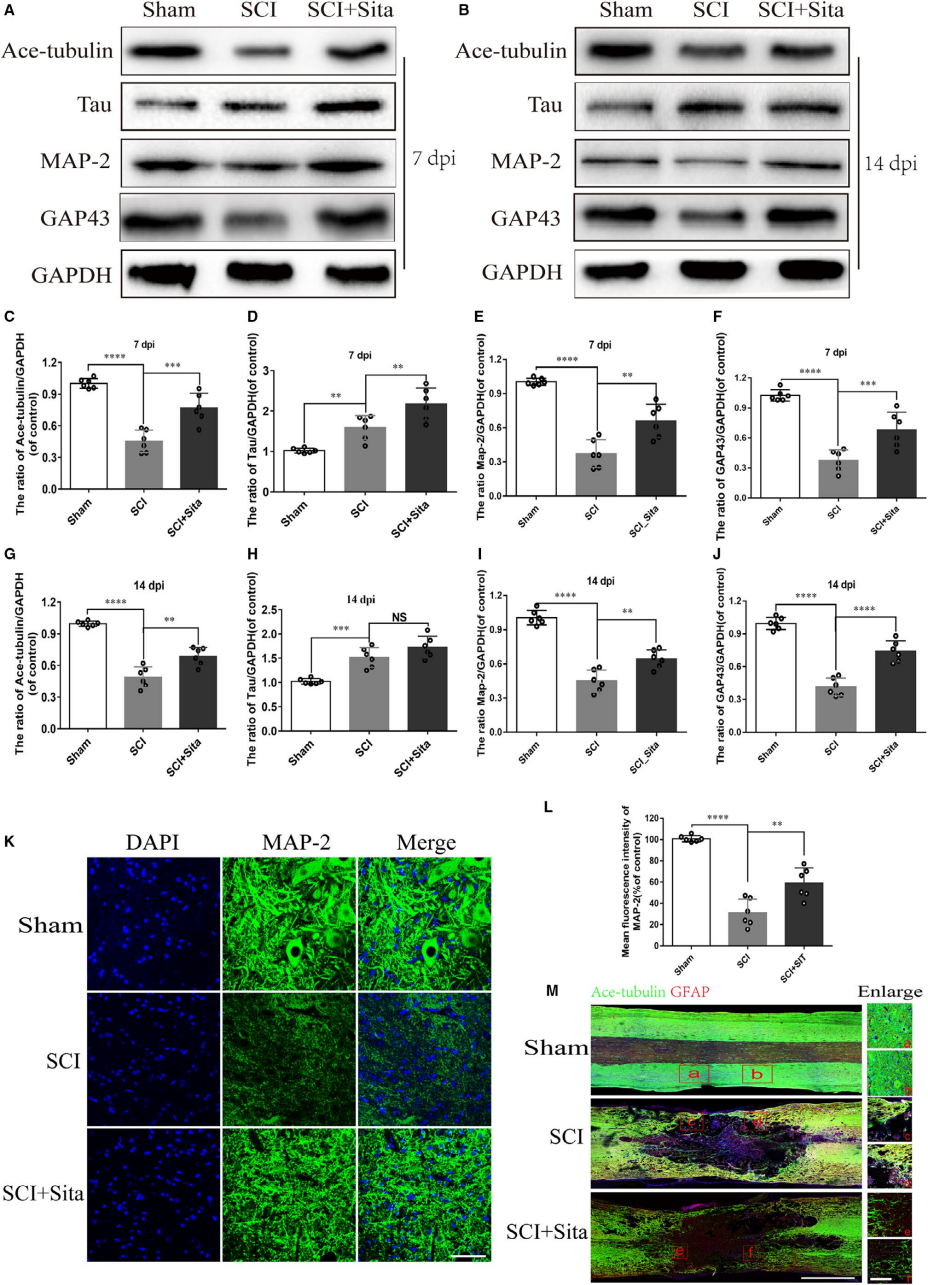
Sitagliptin stabilizes microtubule and facilitates axon restoration after SCI. A‐J, Representative Western blot analysis and quantitative assessment of Ace‐tubulin, Tau, MAP2 and GAP43 expressions at 7 dpi and 14 dpi. K‐L, Immunofluorescence staining and quantitative analysis of MAP2 expression in each group. Scale bar = 50 μm. M, Immunofluorescence staining for Ace‐tub (green) and GFAP (red) in each group at 14 dpi. Scale bar = 1000 μm; scale bar (enlarged) = 200 μm. All data are presented as the mean value ± SEM, n = 6 per group; significant differences among the different groups are indicated by **P < 0.01, ***P < 0.001, ****P < 0.0001. Sham is used as a control

Microtubule (MT) stabilization is essential for axon regeneration, which plays a key role in sensory and motor functional recovery after SCI.^4^ To evaluate the effects of sitagliptin on MT stability and axonal regeneration after SCI, the acetylated tubulin (Ace‐tubulin), a marker of stabilized MT and Map2 as well as GAP43, indicators of axon regrowth were detected at 7 dpi and 14 dpi. Compared with the sham group, the protein levels of Ace‐tubulin, Map2 and GAP43 significantly decreased in the SCI rats. Nevertheless, sitagliptin administration increased these proteins expression after SCI, suggesting that sitagliptin may help microtubules stabilization and axon regeneration after acute SCI (Figure 2A‐J). These results are consistent with that of immunofluorescence with Map2. Compared with sham group, SCI group displayed subtle Map2‐positive axons which were conspicuously enhanced by sitagliptin treatment (Figure 2K‐L). Furthermore, sitagliptin administration improved higher‐density Ace‐tubulin‐positive axons migrating a further distance from the border of lesion area compared against that in SCI group (Figure 2M). Combining all the data mentioned above, sitagliptin may promote stabilization of microtubule and improve axon regeneration.

### 3.4 Sitagliptin reverses SCI‐induced mitochondria dysfunction in vitro

**FIGURE 3:**
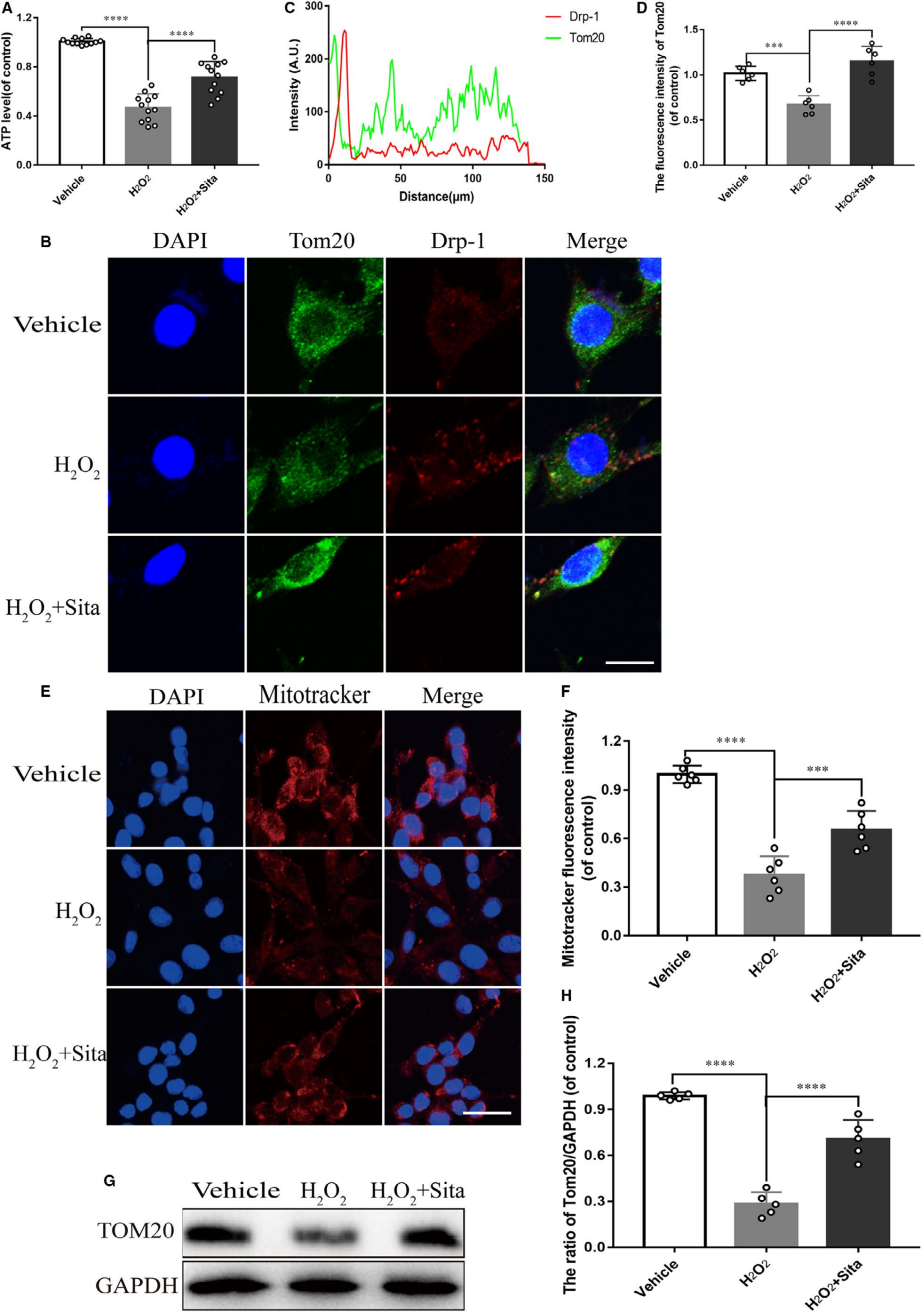
Sitagliptin reverses SCI‐induced mitochondria dysfunction in vivo. A, ATP content was assessed by ATP‐GloBioluminometric Cell Viability Assay. (B‐C) Immunofluorescence double‐labelled staining for co‐localization of Drp‐1 with Tom20 (red: Drp‐1, green: Tom 20, bar: 10 μm). D, The quantitative analysis of fluorescence intensity of Tom20 from (B). E, Mitochondrial membrane potential was detected by Mitotracker and the nuclei were stained with DAPI. (bar = 10 μm). F, The quantitative analysis of Mitotracker fluorescence intensity from (E). G‐H, Western blot and the quantitative analysis of the protein levels of Tom20 in PC12 cells treated as indicated. All data represent mean ± SEM (n = 6 per group). ***P < 0.001, ****P < 0.0001 vs SCI group (or H_2_O_2_ treated group). Sham is used as a control

Previous studies have proved that SCI leads to mitochondrial dysfunction, we therefore explored whether sitagliptin could alleviate the damage of SCI to mitochondria. H_2_O_2_ (100 µmol/L/mL) was employed to simulate SCI microenvironment in PC12 cells, and the optimal concentration of sitagliptin (1 mmol/L) was determinate using Cell Counting Kit‐8 (CCK8) (Figure S2A‐B). The result indicated that H_2_O_2_ treatment increased cellular ATP depletion, while sitagliptin treatment partially restored cellular ATP level in H_2_O_2_ treated cells (Figure 3A). Tom20 is a mitochondria outer membrane receptor and Drp‐1 mediates mitochondria fission, which are two typical protein markers of mitochondria integrity. Similar to the findings, the results of double‐labelled immunofluorescence staining of Drp‐1 and Tom20 displayed changes in co‐localization ratio. As shown in Figure 3B‐D,G‐H, Tom20 decreased sharply after exposure to H_2_O_2_, while sitagliptin rescued this deduction detected by Immunofluorescence staining and western blot. Drp‐1 was highly increased and co‐localized with Tom20 to induce mitochondria fission after H_2_O_2_ treatment. However, stagliptin administration reversed the fission process. These findings were further validated by Mitotracker assay (Figure 3E‐F), a membrane potential‐dependent mitochondria staining method, suggesting that sitagliptin is capable of maintaining mitochondria integrity. Taken all together, sitagliptin contributed to offset the mitochondrial dysfunction caused by SCI.

### 3.5 The expression and activation of neuronal GLP‐1R by sitagliptin

**FIGURE 4:**
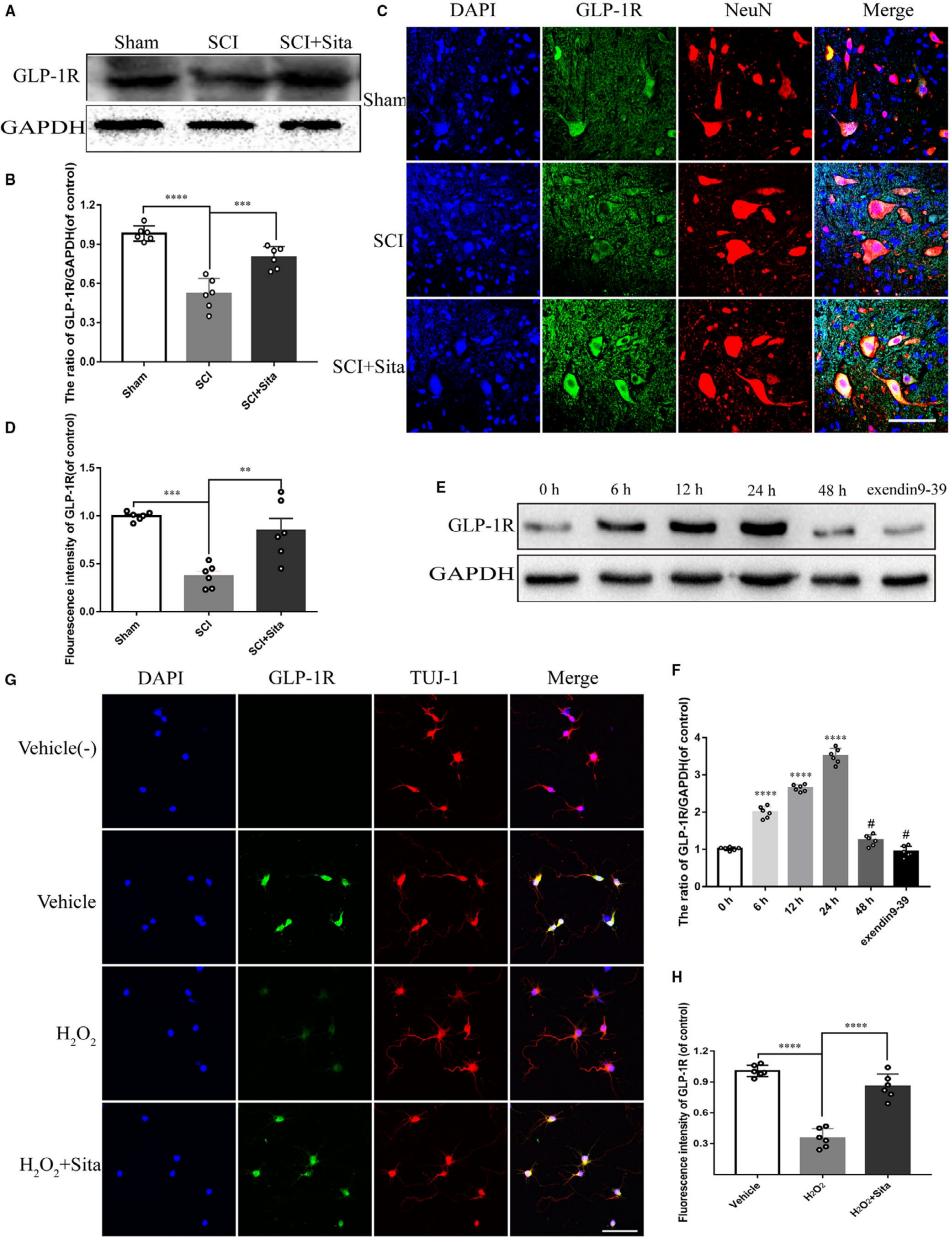
The expression and activation of GLP‐1R by sitagliptin. (A‐B) Western blot and the quantitative analysis of GLP‐1R in SCI rat models treated with or without sitagliptin at 7 dpi. (C‐D) Immunofluorescence double‐labelled staining for GLP‐1R (green) with NeuN (red, bar = 20 μm) and the quantitative analysis of GLP‐1R in each group after SCI. (E‐F) After pre‐treated H_2_O_2_ for 2 h, PC12 cells were treated with 1 mmol/L sitagliptin for 0 h, 6 h, 12 h, 24 h, 48 h, and then Western blot and the quantitative analysis of the protein level of GLP‐1R. (G‐H) Co‐immunofluorescence staining for GLP‐1R (green) with TUJ‐1 (red, bar = 20 μm) and quantitative analysis of GLP‐1R in PC12 cells in each group. All data are presented as the mean ± SEM, n = 6 per group. Significant differences among the different groups are indicated by **P < 0.01, ***P < 0.001, ****P < 0.0001. Sham is used as a control

GLP‐1 targets and stimulates GLP‐1R, regulating various cellular functions. Moreover, GLP‐1R has been approved to distribute widely within spinal cord neurons and gains a great potential for neuroprotection.^21^ Therefore, we hypothesize that GLP‐1R is involved in sitagliptin‐induced neuroprotection. Actually, we observed that the protein expression of GLP‐1R in spinal cord noticeably reduced at 7 days after the trauma injury (Figure S3), while sitagliptin could attenuate the decline of GLP‐1R caused by SCI (Figure 4A‐B). Moreover, the co‐immunofluorescence for NeuN (Red) and GLP‐1R (Green) also showed that the expression of GLP‐1R was up‐regulated by sitagliptin in neurons within spinal cord after injury (Figure 4C‐D). To verify the observation, H_2_O_2_ was employed to simulate the microenvironment of spinal cord injury in PC12 cells and primary cortical neurons isolated from embryonic (E18) foetuses. Western blot analysis exhibited little GLP‐1R in PC12 cells with H_2_O_2_, while the expression of GLP‐1R within sitagliptin‐treated PC12 cells significantly increased in a time‐dependent manner at 6, 12, 24 hours, then sharply decreased at 48 hours (Figure 4E‐F). This reduction of GLP‐1R may be related to degradation of sitagliptin. Similar to the results in vitro, higher fluorescence intensity of GLP‐1R was observed in primary cortical neurons immunostained by Tuj1 in sitagliptin‐treated group compared with H_2_O_2_ group (Figure 4G‐H). Accordingly, sitagliptin stimulates the neuronal GLP‐1R following SCI.

### 3.6 GLP‐1R plays a key role in sitagliptin‐induced locomotor functional recovery

**FIGURE 5:**
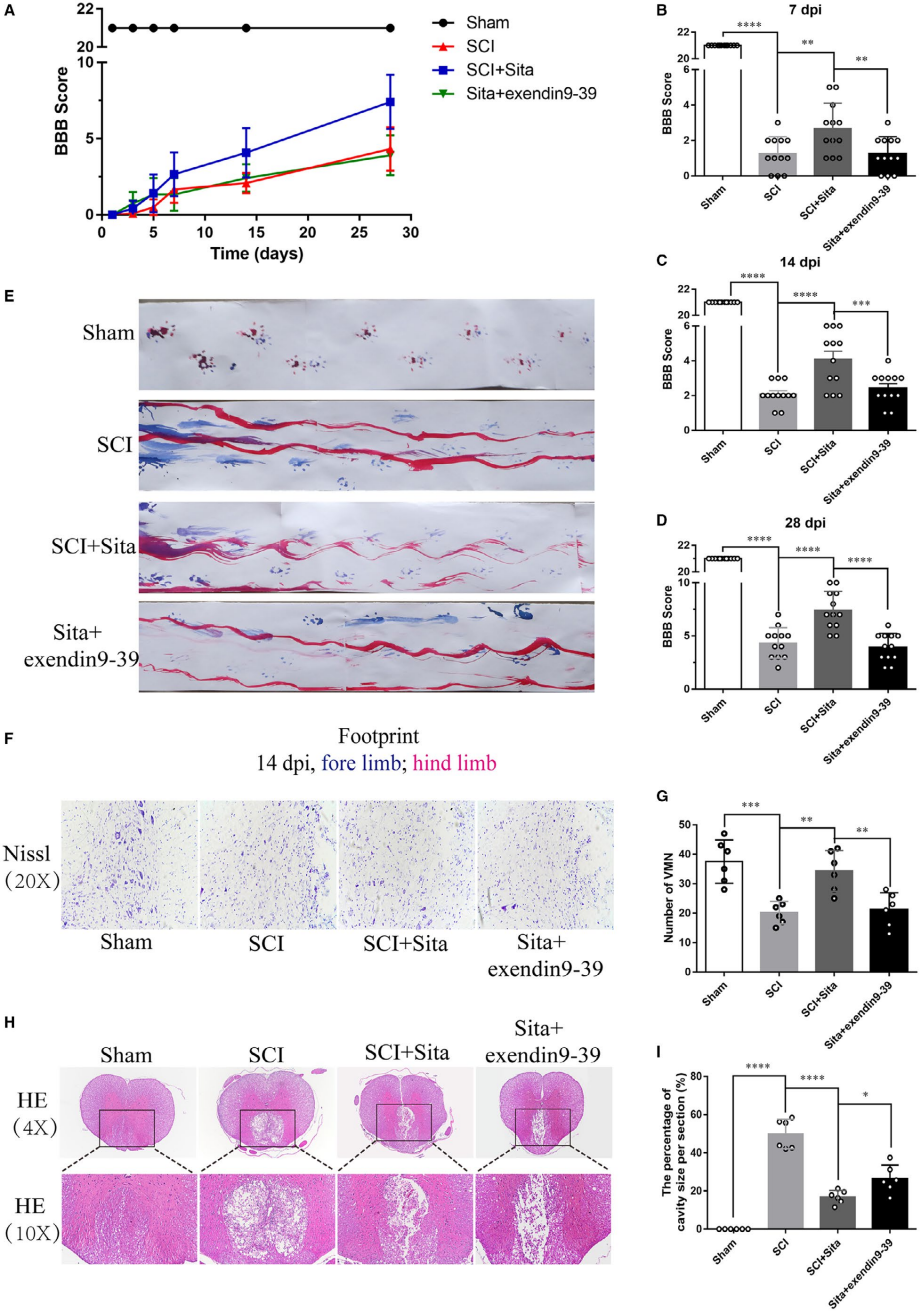
GLP‐1R plays a key role in sitagliptin‐induced locomotor functional recovery. A, The BBB locomotion scores of each group at 1 dpi, 3 dpi, 5 dpi, 7 dpi, 14 dpi and 28 dpi. (B‐D) The statistical analysis of BBB scores at 7 dpi, 14 dpi and 28 dpi from (A), n = 12 per group. E, Footprint analysis in the different groups at 14 dpi (blue: forelimbs; red: hind limbs), n = 6 per group. F, Nissl staining of tissue from each group to evaluate the survival of neurons at 28 dpi. G, The quantification of VMN from (F), n = 6 per group. H, HE staining of spinal cord sections from each group at 28 dpi and (I) the quantification of the percentage of necrotic tissue at each interval, n = 6 per group. All data are presented as the mean ± SEM. Significant differences among the different groups are indicated by **P < 0.01, ***P < 0.001, ****P < 0.0001

GLP‐1R has been approved to play an important neuroprotective role in CNS.^10^ To confirm whether GLP‐1R is key to neuroprotection of sitagliptin following SCI, a selective GLP‐1R inhibitor exendin9‐39 was ip administrated at a dose of 1 μg/kg body weight once daily. As shown in Figure 5A‐E, BBB score and footprint analyses exhibited a dramatic improvement in sitagliptin‐treated group compared with that in SCI, but this trend was inhibited by exendin9‐39, revealing that GLP‐1R contributes to sitagliptin‐induced functional recovery. Moreover, sitagliptin reduced SCI‐induced neuron loss detected by Nissl staining, which was blocked by exendin9‐39 (Figure 5F‐G). Similar to the results, sitagliptin decreased the cavity of necrotic tissue at the injury site while exendin9‐39 administration noticeably counteracted the beneficial effect of sitagliptin (Figure 5H‐I). Overall, these data suggest that GLP‐1R plays a key role in sitagliptin‐induced locomotor functional recovery.

### 3.7 Sitagliptin‐stimulated GLP‐1R mediates the enhancement of nerve outgrowth and axon sprout

**FIGURE 6:**
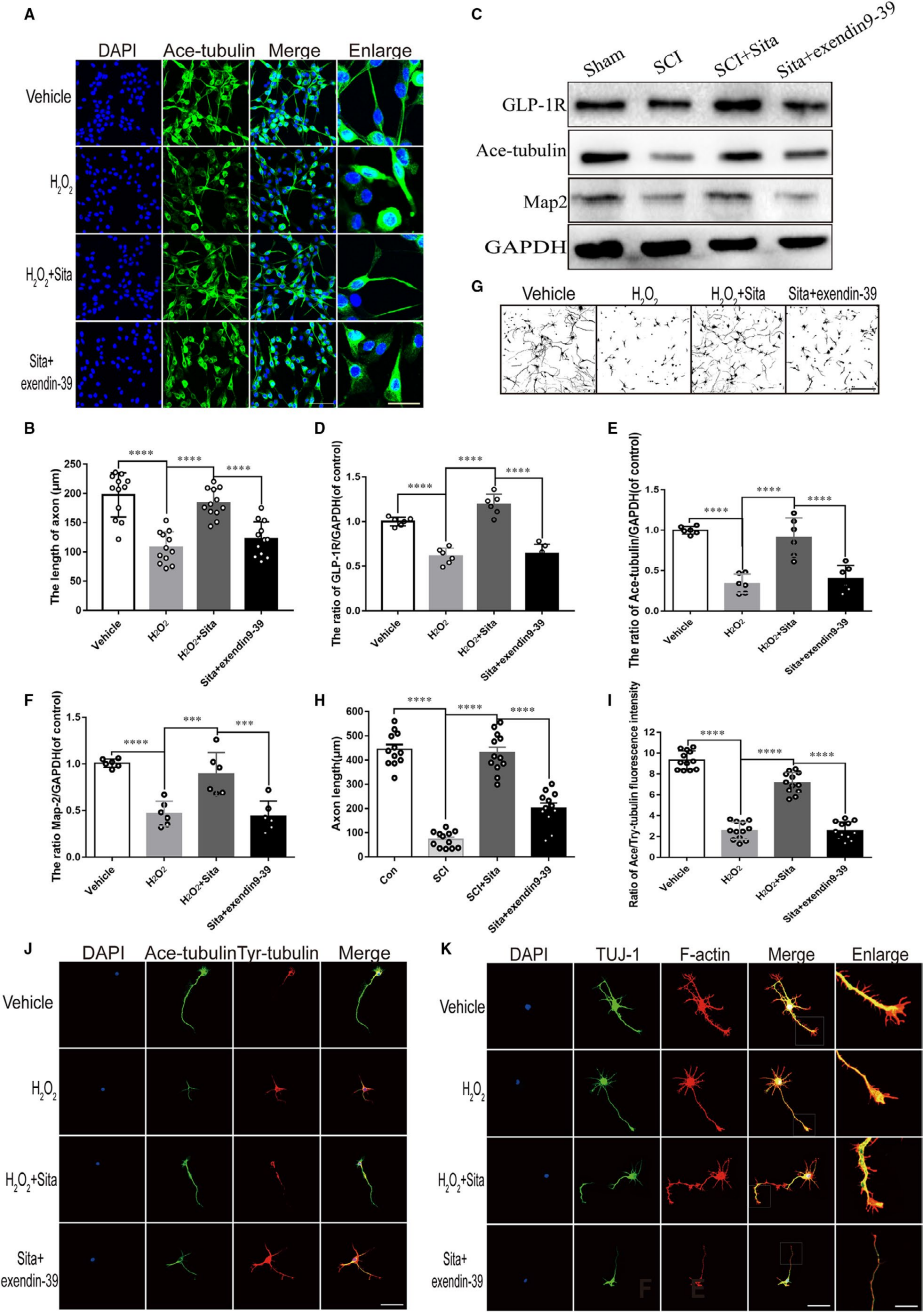
Sitagliptin‐stimulated GLP‐1R mediates the enhancement of nerve outgrowth and axon sprout. (A and B) Immunofluorescence staining for Ace‐tub (green) and the quantitative analysis of axon length in PC12 cells subjected to H_2_O_2_, sitagliptin and exendin9‐39 treatment, scale bar = 50 μm, n = 3 per group. (C‐F) Representative western blot analysis and quantitative assessment of Ace‐tubulin, MAP2 and GAP43 expressions at 7 dpi, n = 6 per group. (G and H) Immunofluorescence staining for Tuj1 and quantification of axon length in primary cortical neurons, scale bar = 100 μm, n = 3 per group. J, Co‐immunofluorescence staining for Ace‐tub (green) and Tyr‐tub (red) in primary cortical neurons in each group after five days culture, scale bar = 50 μm. I, The quantification of the ratio of fluorescence intensity of Ace‐tubulin/Tyr‐tubulin from (J), n = 3 per group. K, Co‐immunofluorescence staining of TUJ‐1 (green) and F‐actin (red) to identify the growth cones of primary cortical neurons in each group after three days culture. Scale bar = 50 μm; scale bar (enlarge) = 10 μm, n = 3 per group. All data are presented as the mean value ± SEM; significant differences among the different groups are indicated by *P < 0.05, **P < 0.01, ***P < 0.001, ****P < 0.0001. Sham is used as a control

To determine the effect of GLR‐1R stimulated by sitagliptin on neurite growth in vitro, PC12 cells and primary cortical neurons were exposed to H_2_O_2_ (1 μmol/L/μL) to simulate SCI microenvironment. As shown in immunofluoresence staining for Ace‐tubulin in PC12 cells (Figure 6A‐B) and for Tuj1 in primary cortical neurons (Figure 6G‐H), both vehicle and sitagliptin‐treated groups showed orderly and longer axons. However, the PC12 cells and primary cortical neurons exposed to H_2_O_2_ alone or exendin9‐39 exhibited curly and shorter axons, exposing that GLP‐1R may favour sitagliptin to promote the axon spout. Similarly, Western blot analyses mirrored an enhancement of GLP‐1R, Ace‐tubulin and Map2 in sitagliptin administrated group compared with SCI group, which was overturned by the GLP‐1R antagonist exendin9‐39 (Figure 6C‐F).

To further verify the role of GLP‐1R in sitagliptin‐induced neurite growth, the primary cortical neurons were co‐immunostained for acetylated (Ace) and tyrosinated (Tyr) tubulins. The ratio of acetylated to tyrosinated tubulin (A/T ratio) represented the relative ratio of stable to dynamic microtubules (MTs). We spotted that treatment with sitagliptin caused a clear increase in the A/T ratio and developed a polarized single and longer axon compared with H_2_O_2_ group, which was impaired by exendin9‐39 (Figure 6I‐J). These results indicate that GLP‐1R stimulated by sitagliptin may promote axonal formation via stabilizing MTs. In addition, the results above were supported by co‐staining of TUj1 (green) and F‐actin (red), a growth cone marker. As shown in Figure 6K, H_2_O_2_ remarkably impaired neurite growth and many of the neurons failed to initiate neurites or only spouted few very short processes compared with vehicle group. Treatment with sitagliptin enhanced neurons neurite outgrowth obstructed by H_2_O_2_, and which was contracted by exendin9‐39. Taken together, these results support that sitagliptin enhances neurite outgrowth and axon sprout by stimulation of GLP‐1R.

### 3.8 Stimulation of GLP‐1R by sitagliptin attenuates neuronal apoptosis and activates AMPK/ PGC‐1α signalling pathway

**FIGURE 7:**
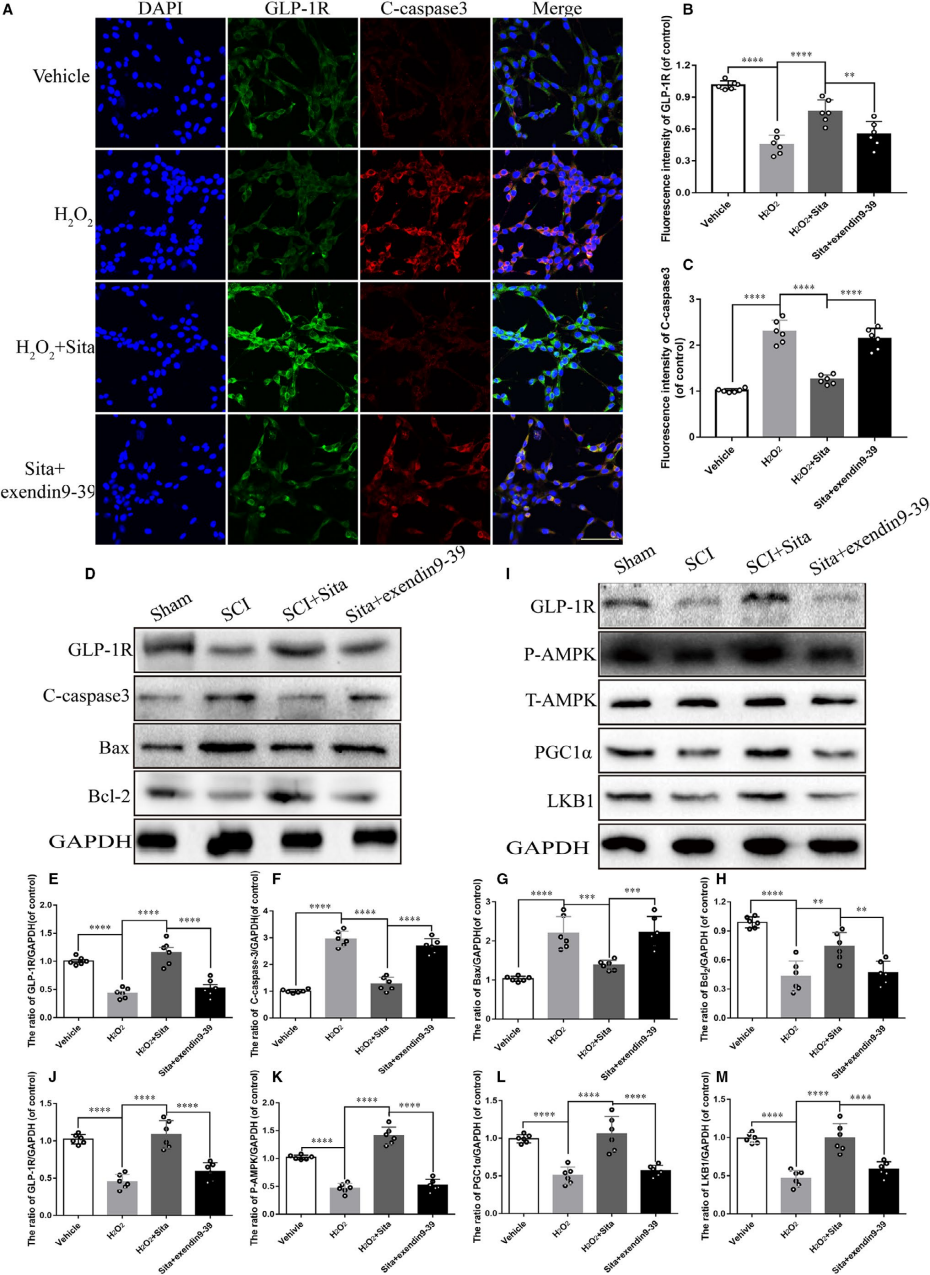
Sitagliptin‐stimulated GLP‐1R reduces PC12 cell apoptosis and activates AMPK/ PGC1α signalling pathway. A, Co‐immunofluorescence staining of GLP‐1R (green) with C‐caspase3 (red). (B and C) The quantitative analysis of fluorescence intensity of GLP‐1R and C‐caspase 3 from (A), n = 5 per group. (D‐H) Representative western blot analysis and quantitative assessment of GLP‐1R, C‐caspase3, Bax and Bcl2 expression in each group at 7 d after SCI, n = 12 per group. (I‐M) Representative western blot analysis and quantitative assessment of GLP‐1R, P‐AMPK, AMPK, LKB1 and PGC1α expressions in each group at 7 d after SCI, n = 12 per group. All data are presented as the mean value ± SEM. Significant differences among the different groups are indicated by *P < 0.05, **P < 0.01, ***P < 0.001, ****P < 0.0001. Sham is used as control

To establish whether sitagliptin alleviates neuronal apoptosis by regulating GLP‐1R under SCI in vivo and in vitro, rats and PC12 cells treated as above, and the apoptotic activity was detected. Compared with H_2_O_2_ or SCI alone group, sitagliptin notably diminished the pro‐apoptotic proteins cleaved‐caspase3 and Bax and enhanced GLP‐1R and the anti‐apoptotic protein Bcl‐2 detected by immunofluoresence staining and western blot, which were reversed by the GLP‐1R inhibitor exendin9‐39 (Figure 7A‐C,D‐H). These results manifest that sitagliptin may attenuate neuronal apoptosis via activation of GLP‐1R. AMPK/PGC‐1α signalling pathway has been proved to play an important role in neurons survival and axon regeneration. To gain insight into the underlying mechanism of sitagliptin‐stimulated GLP‐1R inducing neurons restoration, we examined the activity of AMPK/PGC‐1α signalling pathway. Compared with sham group, AMPK phosphorylation, PGC‐1α and LKB1 decreased after SCI, while application of sitagliptin increased these proteins expression, which was reversed by the GLP‐1R antagonist exendin9‐39 (Figure 7I‐M). Combined, these data indicate that sitagliptin activates AMPK/PGC‐1α signalling pathway by stimulation of GLP‐1R, which participates in the neuroprotection of sitagliptin after SCI.

## 4 DISCUSSION

At early stages of injury, rampant neuronal apoptosis arises, which is a key obstacle to the recovery from the stage of secondary injury. Besides, more and more studies pay attention to the intrinsic axon regeneration capacity. Growing evidences indicate that microtubule assembly and stabilization improves growth cone initiation and axon regeneration after SCI.^4^, ^5^, ^28^, ^29^ In the present study, we assessed the neuroprotective effects of sitagliptin and explored the possible underlying molecular mechanism of the neuroprotection of sitagliptin after SCI both in *vivo* and in vitro. We discovered that administration of sitagliptin attenuated neuronal apoptosis, improved microtubule stabilization as well as axon regeneration, and preserved neurological function by stimulating GLP‐1R after SCI. Additionally, we point out that the beneficial effects of GLP‐1R stimulated by sitagliptin in SCI is involved in activating AMPK/PGC‐1α signalling pathway.

Recently, GLP‐1/GLP‐1R signalling axis is a potential therapeutic target in CNS disease.^8^ Certain studies revealed that GLP‐1R was widely expressed in CNS and within neurons.^30^ However, endogenous GLP‐1 is rapidly degraded by DPP‐4, resulting in the half‐life of GLP‐1 short. Sitagliptin, a highly selective DPP‐4 inhibitor, as an available anti‐diabetic agent, is generally used in clinical treatment of type 2 diabetes,^19^, ^31^ without common adverse effects, relevant drug interactions and cardiovascular risk.^14^, ^22^, ^23^ Evidences have revealed that sitagliptin acts its roles, such as reducing blood glucose level, anti‐inflammation, anti‐oxidative stress and anti‐apoptosis in a GLP‐1/GLP‐1R dependent way.^18^, ^32^, ^33^, ^34^ It is generally believed that neuronal apoptosis is a crucial process that is responsible for neurological impairment after SCI. Increasing evidences show that inhibiting neuronal apoptosis is an effective approach to facilitate neural restoration and functional recovery after CNS injury. In this study, we found that sitagliptin improved locomotor functional recovery and reverses neurological deficit following rat SCI. We further used Bcl‐2, Bax and cleaved caspase 3 as markers to measure apoptotic activation level after SCI. Bax is released upon initiation of apoptotic process, cleaved caspase 3 mediates cleavage of cellular components, and Bcl‐2 prevents apoptosis.^35^, ^36^ Our results showed that sitagliptin administration significantly reduced protein expression of pro‐apoptotic Bax and cleaved caspase 3 and increased anti‐apoptotic protein Bcl‐2, particularly within neurons in rats after SCI, indicating sitagliptin plays a role of anti‐apoptosis in SCI rats. In the lesion site, injured axons often retract or form fragmented degenerative morphologies due to microtubule instability.^37^, ^38^ Thus, remodelling of cytoskeleton structures, such as microtubule stabilization, is crucial for initiating injured axonal regrowth and growth cone outgrowth.^3^ Our data provide evidence that sitagliptin induces both microtubule stabilization and improve axon regeneration after SCI. The role of sitagliptin‐induced microtubule stabilization facilitating SCI recovery is consistent with our previous study which shows that FGF13 improves SCI repairing by stabilizing microtubule and enhancing axon regeneration.^5^

To investigate the mechanism of beneficial effects of sitagliptin on axonal regeneration and neural functional recovery after SCI, we applied a GLP‐1R inhibitor, exendin9‐39. We further found that sitagliptin played roles in reducing apoptosis, promoting axon regeneration, enhancing nerve outgrowth and locomotor functional recovery following SCI via stimulating GLP‐1R (Figures 5, 6, 7). These neuroprotective effects of sitagliptin by increasing GLP‐1R are consistent with that of GLP‐1R agonists, such as exentin‐4, in various CNS diseases.^10^, ^16^, ^24^ Li et al have confirmed that exentin‐4, a GLP‐1R agonist, protects cortical and dopaminergic neurons against degeneration and improved motor function by GLP‐1R stimulation in mouse model of Parkinson disease.^11^ Moreover, our previous study also indicates that increasing expression of GLP‐1R by liraglutide, a GLP‐1 analog, induces autophagy and reduces apoptosis in rat SCI model and neuronal cultures.^21^ As main powerhouses of cells, mitochondria are the energy source used to power virtually all cellular functions. Increased oxidative stress and decreased ATP synthesis cause mitochondrial dysfunction following SCI,^39^ which has been suggested to be crucial for secondary injury and subsequent neuronal cell death.^40^ In our study, acute SCI gave rise to mitochondria dysfunction, measured as decrease of Tom20 protein and ATP product and induction of Drp‐1. However, mitochondria dysfunction caused by SCI was reversed by sitagliptin. Many axon regeneration processes require energy; hence, maintenance of mitochondria function and localization of mitochondria to injured axons both are essential for axon migration and regeneration.^41^ As a result, our data reveal that sitagliptin facilitates axon regeneration and neural functional recovery also possibly by alleviation of mitochondria dysfunction.

AMPK, a conserved serine/threonine kinase, when activated, AMPK triggers a switch from ATP‐consuming anabolic pathways to ATP‐producing catabolic pathways, mediating cellular energy homeostasis.^42^, ^43^ In CNS, activation of AMPK is found to reduce apoptosis and confer neuroprotection in ischaemic stroke.^44^, ^45^ Furthermore, up‐regulation of the AMPK/PGC1α signalling pathway leads to neurite outgrowth and axon regeneration.^46^ The AMPK/PGC1α pathway is regulated by many upstream signalling molecular. Several lines of evidence support the involvement of AMPK in mediating the beneficial effects of GLP‐1 or GLP‐1R.^9^, ^44^, ^47^, ^48^ Our results demonstrated that sitagliptin could activate AMPK/PGC1α pathway. Moreover, we detected that increased GLP‐1R protein level was accompanied by down‐regulation of apoptosis and enhancement of AMPK activity as well as PGC1α protein level when rats treated with sitagliptin after SCI (Figure 7). Thus, these data indicate that GLP‐1R stimulated by sitagliptin facilitating neurons survival is involved in up‐regulation of the AMPK/PGC1α pathway following SCI.

In conclusion, we have provided a combined evidence to demonstrate for the first time that sitagliptin plays protective roles via activating GLP‐1R during SCI recovery. The beneficial effects of sitagliptin stimulating GLP‐1R on SCI repair include anti‐apoptosis, improving microtubsule stabilization and axon regeneration, ameliorating mitochondria dysfunction and finally promoting locomotor functional recovery. Additionally, the helpful role of GLP‐1R induced by sitagliptin is related to AMPK/PGC1α signalling pathway. This study lays a ground work for future clinical studies in sitagliptin or GLP‐1R agonist in SCI recovery.

## CONFLICT OF INTEREST

The authors confirm that there are no conflicts of interest.

## AUTHOR CONTRIBUTION

**Wen Han:** Data curation (equal); Investigation (lead); Project administration (lead); Writing‐original draft (equal). **Yao Li:** Investigation (equal). **Jiangting Cheng:** Investigation (equal). **Jing Zhang:** Investigation (supporting). **Dingwen Chen:** Investigation (supporting). **Mingqiao Fang:** Investigation (supporting). **Guangheng Xiang:** Data curation (supporting). **Yanqing Wu:** Resources (supporting); Software (supporting). **Hongyu Zhang:** Resources (lead); Software (lead). **Ke Xu:** Writing‐review & editing (supporting). **Hangxiang Wang:** Funding acquisition (supporting); Project administration (supporting); Writing‐review & editing (supporting). **Ling Xie:** Conceptualization (lead); Data curation (equal); Project administration (lead); Writing‐original draft (equal); Writing‐review & editing (lead). **Jian Xiao:** Conceptualization (supporting); Project administration (supporting); Validation (equal); Writing‐original draft (supporting); Writing‐review & editing (supporting).

## Supporting information

Fig S1

Fig S2

Fig S3

## Data Availability

The data were used to support the findings of this study are available from the corresponding author upon request.
