# Supplementary figures and images for: Sitagliptin improves functional recovery via GLP‐1R‐induced anti‐apoptosis and facilitation of axonal regeneration after spinal cord injury
Source: J Cell Mol Med. 2020 Jun 22;24(15):8687–702. doi: 10.1111/jcmm.15501 (PMC7412681; doi:10.1111/jcmm.15501)

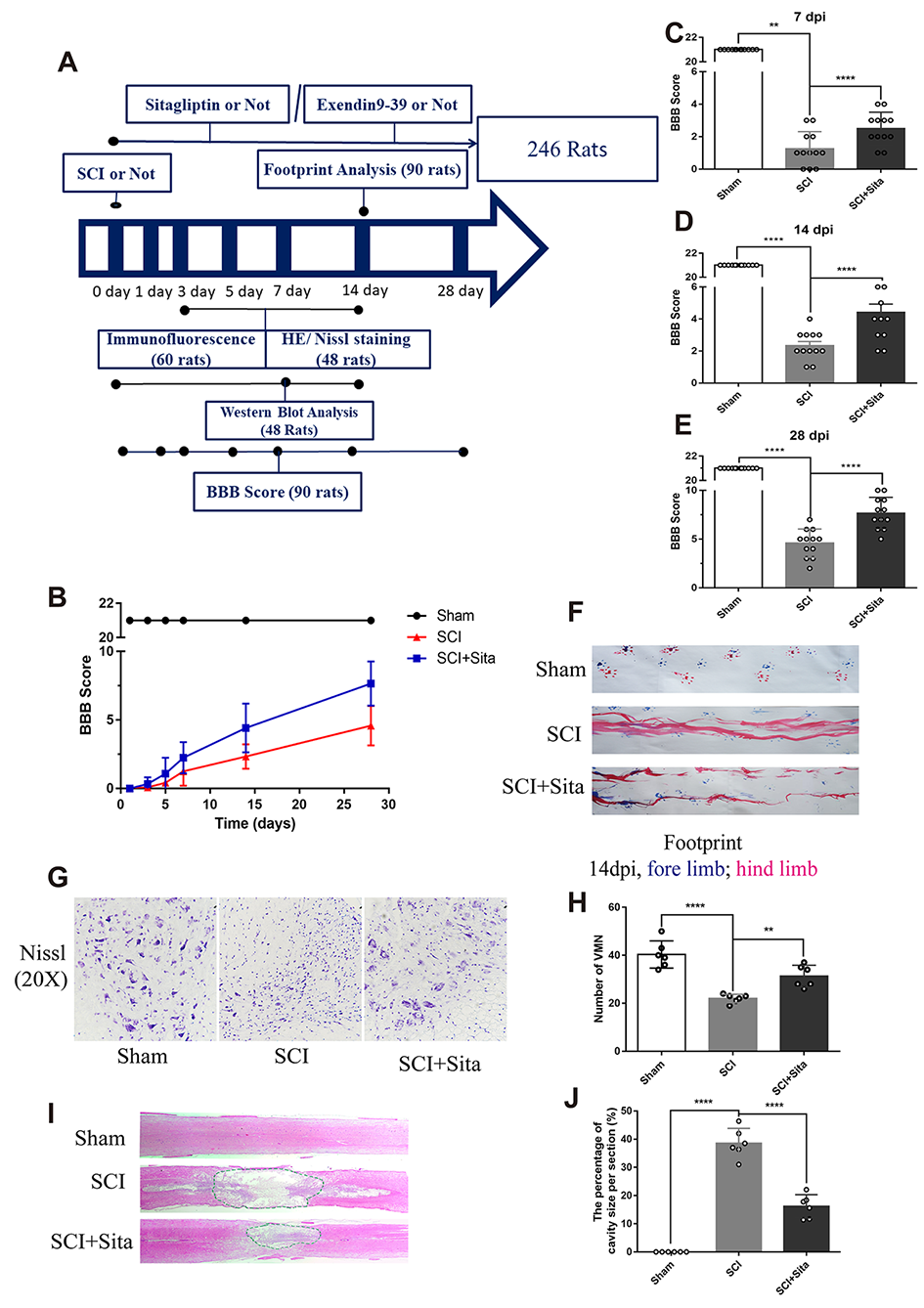

Supplement: Supplementary file 1 — Fig S1 [file JCMM-24-8687-s001.tif]

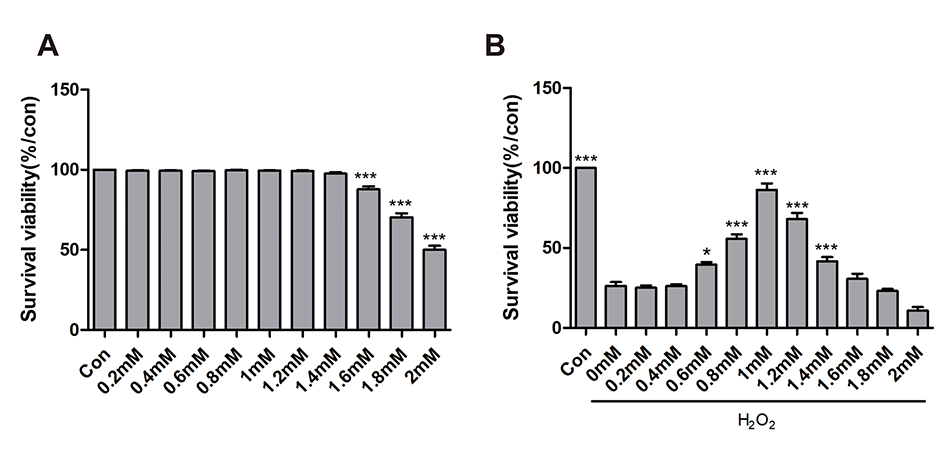

Supplement: Supplementary file 2 — Fig S2 [file JCMM-24-8687-s002.tif]

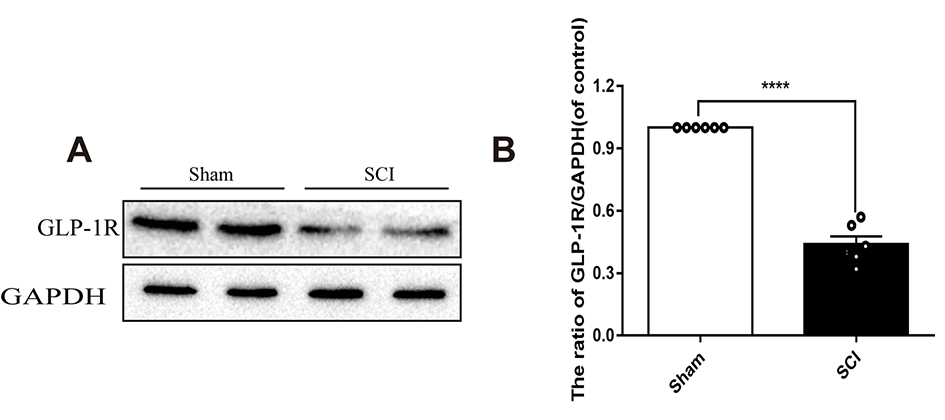

Supplement: Supplementary file 3 — Fig S3 [file JCMM-24-8687-s003.tif]
